# Impact of Physical Activity Interventions on Blood Pressure in Brazilian Populations

**DOI:** 10.5935/abc.20150048

**Published:** 2015-09

**Authors:** Vivian Freitas Rezende Bento, Flávia Barbizan Albino, Karen Fernandes de Moura, Gustavo Jorge Maftum, Mauro de Castro dos Santos, Luiz César Guarita-Souza, José Rocha Faria Neto, Cristina Pellegrino Baena

**Affiliations:** Pontifícia Universidade Católica do Paraná, Curitiba, PR – Brazil

**Keywords:** Motor Activity, Assessment of Health Impact, Blood Pressure, Epidemiology

## Abstract

**Background:**

High blood pressure is associated with cardiovascular disease, which is the leading cause of mortality in the Brazilian population. Lifestyle changes, including physical activity, are important for lowering blood pressure levels and decreasing the costs associated with outcomes.

**Objective:**

Assess the impact of physical activity interventions on blood pressure in Brazilian individuals.

**Methods:**

Meta-analysis and systematic review of studies published until May 2014, retrieved from several health sciences databases. Seven studies with 493 participants were included. The analysis included parallel studies of physical activity interventions in adult populations in Brazil with a description of blood pressure (mmHg) before and after the intervention in the control and intervention groups.

**Results:**

Of 390 retrieved studies, eight matched the proposed inclusion criteria for the systematic review and seven randomized clinical trials were included in the meta-analysis. Physical activity interventions included aerobic and resistance exercises. There was a reduction of -10.09 (95% CI: -18.76 to -1.43 mmHg) in the systolic and -7.47 (95% CI: -11.30 to -3.63 mmHg) in the diastolic blood pressure.

**Conclusions:**

Available evidence on the effects of physical activity on blood pressure in the Brazilian population shows a homogeneous and significant effect at both systolic and diastolic blood pressures. However, the strength of the included studies was low and the methodological quality was also low and/or regular. Larger studies with more rigorous methodology are necessary to build robust evidence.

## Introduction

Cardiovascular diseases are the leading cause of death in Brazil, generating high medical and socioeconomic costs^1^. Hypertension is a highly prevalent risk factor among us, and responsible for approximately 45% of the cases of ischemic heart disease and 51% of those of cerebrovascular disease^2,3^. Lifestyle changes, in particular physical activity and dietary modifications, are the cornerstones of treatment for hypertensive patients, both at primary and secondary levels^4^.

Physical activity reduces the incidence of hypertension in pre-hypertensive individuals, reducing the mortality and the risk of development of cardiovascular diseases^4^. Studies with foreign populations demonstrate that physical activity can lower blood pressure and decrease the prevalence and incidence of hypertension regardless of associated risk factors^5^, in addition to promoting the reduction of blood pressure in patients with resistant hypertension^6^.

Studies analyzing the effects of physical activity on blood pressure levels in the Brazilian population are still scarce^7^. Considering that, the objective of this study was to assess systematically the role of physical activity on blood pressure in the Brazilian population.

## Methods

### Search strategy

We searched electronic databases in health sciences – Medline (Medical Literature Analysis and Retrieval System Online), PubMed (Public Medline), Embase (Excerpta Medica dataBase), The Cochrane Library, CINAHL, Web of Science, SciVerse Scopus, SciELO (Scientific Electronic Library Online), LILACS (Latin American and Caribbean Health Sciences Literature) and VHL (Virtual Health Library) – using a combination of descriptors, including NLM's (US National Library of Medicine) MeSH (Medical Subject Headings) terms text descriptors.

To conduct the systematic review and analyze the methodological quality of the studies, we followed PRISMA (Preferred Reporting Items for Systematic Reviews and Meta-Analyses) guidelines and an extension of the CONSORT (Consolidated Standards of Reporting Trials Statement). We evaluated 27 items required to be reported on a systematic review^8,9^.

The terms used in the search were related to the population analyzed (for example, Brazil* [mesh] OR South America [mesh] OR South America* [tiab] OR Latin America* [tw]), to physical activity interventions combined with the final findings related to blood pressure and hypertension (such as "life style" OR "lifestyle" OR "weight loss" OR "losing weight" OR "weight reduction" OR "disease management" OR "exercise" OR "exercise therapy" OR "exercise test" OR "exercise movement techniques" OR "kinesiotherapy" OR "physical endurance" OR "anaerobic" OR "aerobic" OR "exercise" OR "resistance training" OR "motor activity*" OR "physical activity*" OR "locomotor activity" OR "social support" OR "social network" OR "tobacco use cessation" OR "smoking cessation" OR "alcohol drink" OR "alcohol consum*" OR "drinking alcohol" OR "alcoholi*" OR "non-pharmacol*") AND ("blood pressure" OR "hypertension"), and type of selected studies ("randomized" OR "placebo" OR "randomly" OR "trial" OR "groups" OR "comparative" OR "evaluation" OR "effectiveness" OR "utility" OR "validation" OR "reliability"). References cited in articles identified by the search strategy were also searched manually and added up to the study and the literature review. The searches were carried out until May 14, 2014.

### Inclusion and exclusion criteria

Regarding the design of the study, we included randomized clinical trials, clinical trials, community studies with comparison of an intervention group with a control group, studies conducted with adult individuals, studies reporting (systolic and diastolic) blood pressure levels in the same cohort before and after the intervention in the control and intervention groups, and studies analyzing the effect of physical activity interventions on blood pressure.

We excluded studies and reports developed outside Brazil, those with interventions involving drug therapy, studies including pregnant women, animal studies, those with interventions shorter than eight weeks, letters, abstracts, conference proceedings, and observational, *crossover* and conglomerates studies.

### Study identification and selection

**Figure 1:**
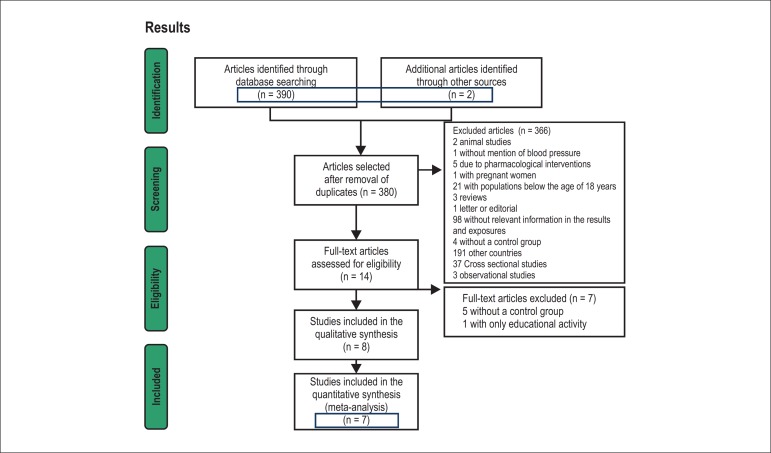
Flowchart of the selection process of the studies.

Two pairs of authors read separately and independently the titles and abstracts of each pre-selected study to identify those that fulfilled the inclusion criteria. Following that, the articles were read separately by four authors to ensure that the criteria of the systematic review were met. Disagreements between the authors were resolved by discussion and dialogue in the presence of a fifth author. The selection of the studies included in the systematic review was then finalized and those meeting the criteria for the meta-analysis were identified (Figure 1).

### Data extraction

Two authors collected the data in a predefined form. A third author reviewed the extracted data independently. The characteristics of the extracted studies included, among others, date of publication, title, study definition, intervention duration, type of intervention and supervision. We registered the information about the participants in each study, the number of participants including the total number of participants in the analysis, gender, age, area of residence (whether urban or rural), use of medications and comorbidities. Finally, we collected the results related to blood pressure before and after the intervention with their respective variances.

The quality of each study was evaluated by the Cochrane Collaboration's tool for assessing risk of bias^10^, which contains the following criteria: sequence generation, allocation concealment, blinding of participants, blinding of results and outcome assessors, integrity of the results, incomplete data, selective outcome reporting and other sources of bias (for example, the number of participants).

### Statistical analysis

Both systolic and diastolic blood pressures were recorded as continuous variables in mmHg. The effect size of each study was calculated as the difference of the pre-and post-intervention mean measurements in the intervention group minus those in the control group. When absent, the variances of the pre-and post-intervention differences in the intervention and control groups were imputed following a methodology described previously^11^. All analyses were performed using the software Stata Corp LP, College Station, Texas, USA, considering a significance level of 5%.

For the meta-analysis, we used fixed and random effects models with a 95% confidence interval (CI). To analyze the heterogeneity of the studies, we used the I^2^ test^12^.

Publication bias was assessed with a funnel plot. The effect of small studies was assessed with the Egger test^12^.

## Results

### Identification and selection of the studies

Of the 390 references retrieved with the search strategy, 14 full-text articles were obtained for reading. Five were then excluded due to lack of a control group, one due to the absence of intervention and another for not presenting measures of variance. Finally, eight studies fulfilled the inclusion criteria proposed for the systematic review and seven for the meta-analysis (Figure 1).

### General characteristics of the selected studies

The main characteristics of the studies included in the systematic review are shown in Table 1. Considering only the studies selected for the meta-analysis, the samples ranged from 19 to 177 participants with a total of 493 participants with an average age of 61.8 years and standard deviation of 9.7 years. Two studies evaluated only women^13,14^, and the remaining studies included individuals of both genders. Among these, one^15^ reported a greater proportion of men, whereas all others reported greater proportions of women. Regarding the occurrence of comorbidities, three articles did not report the occurrence of associated pathologies^15-17^, one of the studies evaluated only patients with type 2 diabetes mellitus^14^, and another reported patients with osteoporosis^13^. The remaining studies included individuals with metabolic syndrome or at least one of its components (diabetes, hypertension or obesity). The average duration of the interventions was 16.9 weeks with a standard deviation of 7.5 weeks. The quality of the studies evaluated according to the Cochrane tool^10^ is shown in Table 2. None of the selected studies had analysis based on an intention to treat.

**Table 1:** Characteristics of randomized clinical trials included in the systematic reviewM

**First author **	**Year **	**Sample size **	**Mean age (years), gender**	**Comorbidities **	**Intervention **	**Duration (weeks)**
Terra et al.^13^	2008	46	66.8, F	Diabetes, osteoporosis, dyslipidemia	Resistance exercise, 3 sessions per week	12
de Meirelles et al.^23^	2009	19	49, FM	Hypertension, cardiovascular disease, diabetes	60-minute exercises, 3 sessions per week	12
Bündchen et al.^24^	2010	111	58, FM	BMI > 30 (49.2%)	Aerobic and resistance exercises, 3 sessions per week Walking, water aerobics,	12
Vianna et al.^16^	2011	70	69.8, FM	No	stretching and resistance exercise, 3 sessions per week	16
Kanegusuku et al.^15^	2011	24	63 M/F	No	Resistance exercise, 2 sessions per week	16
Barroso et al.^25^	2008	24	66.5, M/F	Hypertension	60-minute exercises, 3 sessions per week	18
Monteiro et al.^14^	2010	22	F	100% T2DM	50-minute aerobic training, 3 sessions per week 150-minute exercises of	13
Cezaretto et al.^17^	2011	177	M/F	No	moderate physical activity per week	36

Note: M: male; F: female; T2DM: type 2 diabetes mellitus; BMI: body mass index.

**Table 2:** Characteristics of the randomized clinical trials included in the systematic review

**First author **	**n **	**Control Group **				**n **	**Intervention Group **			
		**Systolic **		**Diastolic **			**Systolic **		**Diastolic **	
		**Pre **	**Post **	**Pre **	**Post **		**Pre **	**Post **	**Pre **	**Post **
de Meirelles et al.^23^	6	141.7 (6)	145 (6)	91.6(-2)	95 (-2)	13	139	116	89	79 (2)
Barroso et al.^25^	13	141.7	147.5	88.4	94.1	22	145.3	139.9	89.8	85.9
Vianna et al.^16^	35	141.14 (17.95)	142.57 (18.04)	82.29 (7.70)	87.43 (9.50)	35	142.27 (18.32)	132.86 (14.47)	81.43(6.01)	79.43 (6.39)
Cezaretto et al.^17^	80	135.8	136.2	80.5 (9.9)	80 (8.2)	97	136.4 (17.7)	131 (17)	84 (10.7)	76.8 (12.5)
Bündchen et al.^24^	54	139.3 (14)	138.8 (15)	86.1 (9)	86 (-9)	57	145.2 (16)	127.7 (17)	89.3 (12)	81.2(8)
Terra et al.^13^	20	124.6 (10.1)	123.3 (13.5)	74.2 (7.3)	73.3 (7.5)	26	125.2 (9.3)	114.7 (9.2)	72 (6.8)	71.04 (7.9)
Monteiro et al.^14^	11	139.8 (19.53)	128.1 (25.92)	77.5 (10.64)	69.1 (9.83)	11	140 (14.35)	124.5 (19)	75.4 (13.37)	54.4 (3.61)
Kanegusuku et al.^15^	11	124.4 (2.1)	118(3)	78.3 (1.2)	73	15	120.8 (2.4)	117 (-4)	77.9 (1.5)	73 (-3)

Note: The results in parentheses are expressed as mean values (± SD).

### Effects of physical activity on blood pressure

**Figure 2:**
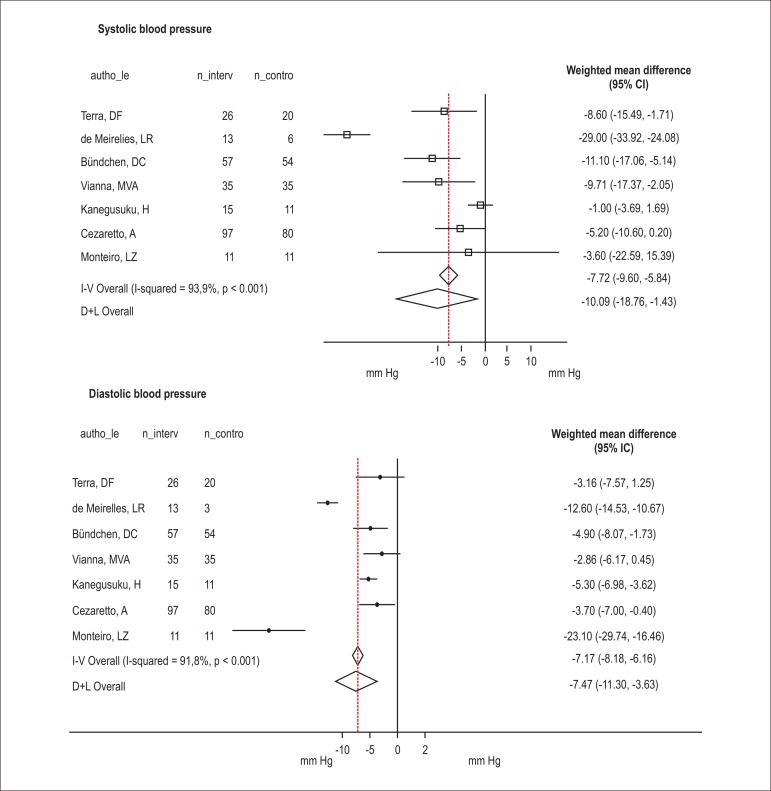
Meta-analysis of the effects of physical activity intervention on systolic and diastolic blood pressures in the Brazilian population.

**Figure 3:**
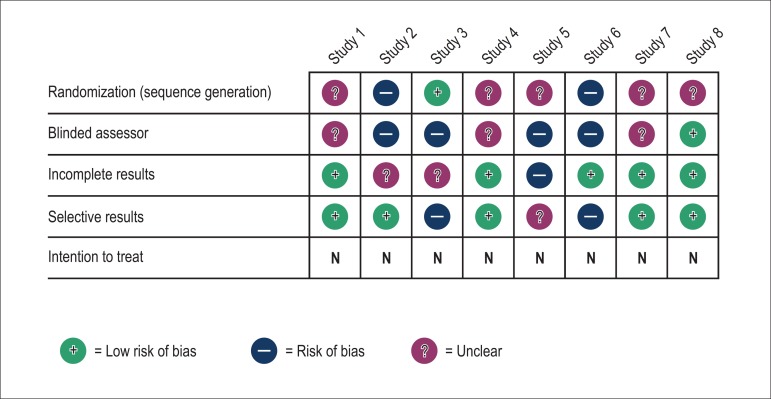
Evaluation of the risk of publication bias – Cochrane tool (adapted).

**Figure 4:**
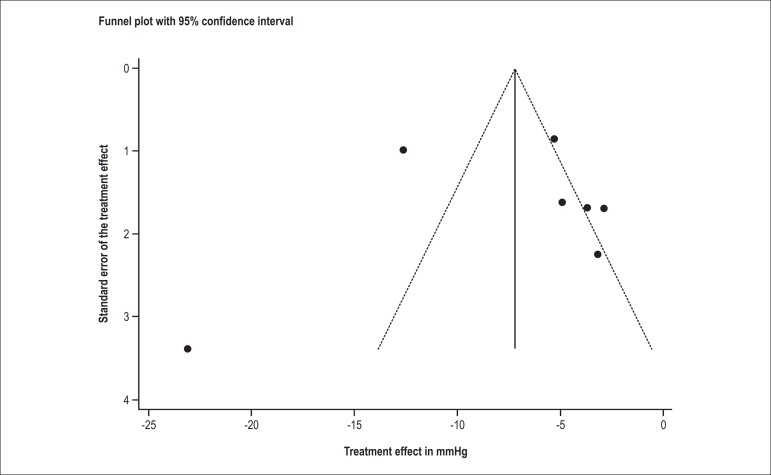
Funnel plot of the studies included in the meta-analysis.

All studies were randomized clinical trials and the assessment of the intervention effect on blood pressure (in mmHg) was performed evaluating the variation in systolic and diastolic blood pressures in the control and physical activity intervention groups (Figure 2) The studies showed heterogeneity in the evaluation of systolic (I^2^ = 93.9%, p < 0.001) and diastolic (I^2^ = 91.8%, p < 0.001) blood pressures. A publication bias was identified by the (adapted) Cochrane tool (Figure 3) and funnel plot (Figure 4). The Egger test showed a small-study effect (p < 0.001).

The physical activity interventions in the analyzed studies included resistance and aerobic exercises. The combined effect of these studies was protective, reducing both the systolic (intervention effect = -10.09, 95% CI: -18.76 to -1.43, I^2^ = 93.9%, p < 0.001) and diastolic (intervention effect = -7.47, 95% CI: -11.30 to- 3.63, I^2^ = 91.8, p < 0.001) blood pressures.

## Discussion

This meta-analysis following a systematic review included seven studies with 493 participants (eight studies were included in the systematic review). We found a heterogeneous effect of physical activity interventions on blood pressure in this population. Factors relevant to this result are the presence of different comorbidities in the studies, as well as different types of intervention, ranging from resistance to aerobic exercises.

The population in this study showed a reduction in blood pressure, demonstrating a statistically significant decrease in both systolic and diastolic pressures. However, since the selected studies had small sample sizes, it is not clear whether they would show the same result had the interventions lasted longer. Similar results were found by Kelley et al. and Cornelissen et al. who evaluated the effectiveness of isometric handgrip exercises and resistance exercises in reducing systolic and diastolic blood pressures. They found reductions in both systolic and diastolic pressures, but generalization of the results was limited due to the small number of studies included^18,19^.

The studies analyzed include individuals with and without comorbidities. Therefore, it is not clear if the effect on specific populations, such as those with hypertensive individuals, would be similar or more protective than the results presented in this meta-analysis.

Regarding the effects of physical activity on blood pressure, the magnitude of blood pressure reduction showed variation when we analyzed the results of other meta-analyses, but physical activity interventions showed a consistent protective effect. As an example, Hagberg et al.^20^ showed a reduction of 11 mmHg and 8 mmHg in systolic and diastolic blood pressures, respectively. The study of Halbert et al.^21^ reported that aerobic physical training reduced in 4.7 mmHg the systolic blood pressure and in 3.1 mmHg the diastolic blood pressure. Finally, a meta-analysis by Whelton et al.^22^ that included 54 controlled studies showed a reduction of 3.7 mmHg and 2.6 mmHg in systolic and diastolic blood pressures.

The distribution of the studies in the funnel plot indicated a risk of publication bias in those included in the meta-analysis. When assessed separately with the Cochrane tool, most f the studies showed unclear and/or high risk of bias. In addition, the Egger test showed a small-study effect in the results.

Some limitations of this meta-analysis should be considered. The first limitation is the quality of the studies (Figure 3). In addition to the data described in the adapted Cochrane table, some studies failed to report basic information such as mean age, socioeconomic variables and presence or absence of comorbidities. The second limitation was the size of the samples, which ranged from 19 to 177 participants.

Strengths of this meta-analysis are the inclusion of only randomized clinical trials, the absence of restrictive search for publications only in English, and the assessment of the effects of each physical activity intervention regardless of their results.

This study has some implications. The combination of the evidences from available studies allows identification of new research opportunities and points out a need for new scientific studies involving these populations, including high quality studies with larger numbers of participants and lasting more than 16 weeks.

## Conclusion

This meta-analysis gathered information about the Brazilian population and showed that physical activity reduced blood pressure levels in the studied population.

The combination of these studies showed a significant decrease in systolic and diastolic blood pressures with the performed interventions, but the strength of the studies analyzed is low and the quality of the methodology is also low and/or regular.

Blood pressure changes promoted by physical activity have been extensively studied. However, they are still little explored in populations of developing countries like Brazil. This gap seen in our country, which has a high prevalence of risk factors for the development of cardiovascular diseases, has as a consequence the development of few programs focused on prevention and reduction of risk factors.

The results of this meta-analysis show a need for studies with longer lasting interventions assessing the influence of physical activity on blood pressure, and for caution regarding the methodology used for randomization of the groups and blinding of assessors to ensure stronger studies with better quality. Such studies will support health care policies directed to hypertensive patients (secondary care), as well as primary prevention of hypertension in individuals with normal blood pressure.
